# American Dental Association

**Published:** 1886-09

**Authors:** 


### THE DENTAL REGISTER.

VOL. XL.] SEPTEMBER, 1886. [No. 9.

### PROCEEDINGS OF SOCIETIES.

### American Dental Association.

TWENTY-SIXTH ANNUAL MEETING, NIAGARA FALLS, AUGUST 3, 4, 5, 6, 1886.

(Reported for DENTAL REGISTER by "Mrs. M. W. J.")

The members of the American Dental Association were called to order in twenty-sixth annual session, at 11 A. M., Tuesday, August 3, 1886, the President, W. C. Barrett, M.D.,D.D.S., Buffalo, N. Y., in the chair.

OFFICERS.

President, W. C. Barrett, Buffalo, N. Y
First Vice-President, L. C. Ingersoll, Keokuk, Iowa.
Second Vice-President, A. T. Smith, Minneapolis, Minn.
Recording Secretary, George H. Cushing, Chicago, Ill.
Corresponding Secretary, A. W. Harlan, Chicago, Ill.
Treasurer, George W. Keely, Oxford, Ohio.

EXECUTIVE COMMITTEE.

J. N. Crouse, Chairman. A. M. Dudley, Secretary.

FIRST DIVISION—COMMITTEE OF ARRANGEMENTS.

J. N. Crouse. L. D. Shepard. W. N. Morrison.

SECOND DIVISION—ON CREDENTIALS, ETHICS, AND AUDITING ACCOUNTS.

A. M. Dudley. S. G. Perry. A. O. Hunt.

THIRD DIVISION—ON VOLUNTARY ESSAYS.

C. N. Pierce. Geo. J. Friedrichs. A. H. Thompson.

LOCAL COMMITTEE OF ARRANGEMENTS.

S. A. Freeman, Buffalo, N. Y. Geo. L. Field, Detroit, Mich.
J. W. Wassall, Chicago, Ill.

The opening proceedings were somewhat shorn of their usual grave dignity by the calling to order of the President by the Treasurer, Dr. Geo. W. Keely, Oxford, Ohio.

No member has a right to call the President to order, or to call any member to order. He may rise to a point of order, said the President.

I rise to a point of order, Mr. President.

State your point of order, Mr. Treasurer.

According to the rules of this Association, no one has a right to the floor until he has paid his dues!

The President joined heartily in the laugh at his expense, and paid his dues promptly, and the business of the meeting proceeded in due form.

The roll was called and 104 members found to be present.

The reading of the minutes of the last annual meeting, held at Minneapolis, was dispensed with, as they are in the hands of the members in published form.

The first item of miscellaneous business was the resolution offered at the last meeting, changing the Constitution, Article IV., third line, striking out first and substituting the fourth Tuesday in August, as time of meeting. A motion to lay on the table was carried by a large majority.

The consideration of a further Amendment to the Constitution, leaving the selection of the place of meeting to the Executive Committee, was, on motion of the original mover of the resolution at the last meeting (Dr. C. N. Pierce) indefinitely postponed.

*Dr. A. H. Thompson* (Topeka, Kan.) offered a resolution amending Section 2 of Article VI. of the Constitution, so as to allow the members to join two or more sections, instead of only one, and also obliging them to declare their choice of sections at the time of paying dues and joining the Association.

*Dr. J. N. Crouse* (Chicago) said that it was so difficult to get members to work in one section, that it would be useless to expect them to work in more than one.

*Dr. Thompson* thought it would con.er a benefit by distributing the real workers among different sections instead of restricting each worker entirely to one section.

*Dr. J. S. Marshall* (Chicago) thought there should be no restrictions placed upon members as to the amount of work they might perform. A man might want to present two papers in different sections. The sections should be open to all members.

*Dr. Geo. H. Cushing* (Chicago) said that point was met by the fact that volunteer papers were referred to whatever section they pertained, the writer having access to that section with his paper for the purpose of debate. That, however, did not give him a voice in the organization of the section, or the election of its officers.

After some further discussion the resolution was temporarily laid on the table.

The Committee of Arrangements offered their printed programme as a partial report, which was accepted.

The Committee on Publication reported that the transactions of the last annual meeting had been published as usual by the S. S. White Co. without cost to the Association. They were substantially bound in cloth, as per resolution, and had been mailed to all members in December, a much earlier date than usual. The committee desired to express their obligations to Mr. Hice for his valuable assistance. They reported that two manuscript copies of the proceedings of 1883 had been made for the Congressional Library at Washington as per resolution. The committee presented an itemized statement of receipts and expenditures, showing a balance due the committee of $21.40.

The report was received and referred to the Auditing Committee.

The Treasurer asked for further time.

*Dr. Metcalf* (Kalamazoo) stated that on presenting credentials as delegate from the Michigan State Dental Association, he had been met with the statement that there was $10.00 back dues charged against him on the books of the Treasurer. That he had never been a permanent member, but that whenever he had attended as delegate, he had paid his dues. He wanted the matter examined into and explained, as he was not aware of any indebtedness on his part. He was willing at any time to contribute to the support of the Association, but was not willing to pay unjust charges.

*Drs. Taft, Allport,* and others thought that if a member had not been duly notified at the proper time, there was no indebtedness.

*Dr. Crouse* said that no man could be received as delegate who owed back dues. That Societies could not dictate to the Association whom it should receive as delegate, if the rules of the Association prevented admission.

*Dr. W. W. Allport* said that a man could not be charged with dues unless he had signified his intention of becoming a permanent member. A delegate legally authorized by a Society could not be rejected by the Association.

*Dr. Metcalf* said as far as legality went, there was no legal debt as it had long ago expired by limitation if it had ever been due, which, however, was not the case.

*Dr. Crouse* rose to a point of order. Dr. Metcalf, not having been received as a member, had no right to the floor.

The Treasurer explained that when the books came into his hands he had carried into his new set of books all the dues he found unpaid. The dues of 1875 and 1876 were charged to Dr. Metcalf, and cannot be dropped till paid. He cited the case of Dr. C. M. Wright, who went to Europe, and was gone ten years. When he returned he paid $10.00 for the two years due when dropped for non-payment of dues, and $5.00 due for the current year.

*Dr. Taft* stated that unless the records showed that Dr. Metcalf had signified his intention of becoming a permanent member there could be no indebtness on his part.

On motion, the subject was referred to the committee on credentials.

The Treasurer submitted the following report:

| | |
|---|---|
| Balance on hand August, 1885 | $1,948 95 |
| Received of W. R. Clifton | 200 00 |
| Dues received at Minneapolis | 1,020 00 |
| Dues collected during the year | 240 00 |
| Total | $3,408 95 |
| Disbursements | 789 85 |
| Cash on hand | $2,619 10 |

This gratifying financial exhibit was greeted with an outburst of applause and referred to the Auditing Committee.

The President then delivered his Annual Address, which was a polished and scholarly production.

He said that it was his ambition to have the American Dental Association become the foremost one in all the world, and accordingly he would devote himself to the designation of some of the elements in convention which would tend in this direction. The Association should be a dignified body as is befitting the representative men of a profession gathered for an earnest purpose. It should be a scientific body and its conclusions in its special field of investigation should be regarded as final by the remainder of the world. The attitude of each member should not be that of self-assertion, but rather an earnest seeking after light. The convention should discuss only principle and fundamental laws, never descending to trivialities and special methods. The members should study the topic assigned the sections and come prepared for intelligent discussion. Enough work should be laid out in advance for a whole year. Valuable time should not be wasted in haggling over parliamentary points, the will of the majority being regarded as paramount to any established rules for the government of deliberative bodies. And finally, the President warned the members not to be carried away by the wit of the clown. Empty rhetoric, amusing incidents of office practice, poetry and the like should not be applauded and given all the attention, while the dignified and weighty essays giving the results of patient, thoughtful, searching investigation are read to empty chairs.

He hoped that the members would be careful to observe all the amenities of debate, avoiding all personal controversies, private conversation, or any thing else which would interfere with the objects of the meeting; that each one would do all that he could to materially advance the cause all have at heart.

Dr. Dorrance moved that the Address be placed in the hands of the Publication Committee, and spread on the minutes. Carried. On motion, adjourned.

Immediately after adjournment the various scientific sections met for organization.

### FIRST DAY—SECOND SESSION.

Called to order at 8:15 P. M. The President in the chair. The minutes of the morning session were read and approved.

*Dr. Thompson* moved that the Amendments to the Constitution, which were temporarily laid on the table at the morning session, be now called up and opened for discussion. Carried.

*Dr. Thompson* again read the proposed Amendment, which had received some verbal alterations, and which, as re-written, provides that each member may join one or more sections, and that at the roll-call on the second day, each member shall answer to his name by giving the number of the section or sections of his choice.

The resolution was adopted as read, and the Constitution so amended.

There being no further miscellaneous business, the sections were called in proper order.

None were yet ready.

A motion to adjourn was lost.

On calling the roll of sections a second time, Dr. A. H. Thompson, Secretary of Section VII.—Physiology and Etiology, announced a list of papers which had been accepted by the section, which was received as their report. The writers of the papers were not ready to read.

Dr. W. B. Ames, Secretary of Section I.—Prosthetic Dentistry, Chemistry, and Metallurgy, announced his readiness to report.

The President suggested that the doors be closed, with no admittance during the reading of papers or scientific discussion.

Objected to by Dr. Crouse, as excluding members.

Secretary Ames announced that the section had received and approved two papers, one from Dr. W. H. Truman, on Improvements in Vulcanizing and Vulcanizers; and one from Dr. Haskell on Needed Improvements in Mineral Teeth. They also wished to call attention to a valuable paper read before the Kansas Dental Society; and another read before the Connecticut Valley Association. They called attention to the great improvements in carved porcelain blocks, and to the more general use of continuous gum work, due to the improvements in small furnaces, to the skill and ingenuity developed in crown and bridge-work, various small appliances used in the mouth, as separators and matrices. They described the method of Dr. Stockton—a removable piece overcoming the great objection of uncleanliness in permanent fixtures; also the method of Dr. Swasey, in which the teeth are attached to a continuous band answering the same purpose as a very narrow plate, with greater stability than open clasps; also the method of Dr. Melotte, on which clinics will be given; a special material for taking impressions, composed of Potter's clay and glycerine, and a new fusible metal composed of 16 parts bismuth, 10 parts tin, and 6 parts lead; also a new alloy for solder. Dr. Dorrance also has a report to read, embodying a continuation of a former report on the recorded effects of wearing vegetable plates. The section requested that this report be considered before the papers were read.

*Dr. Wm. H. Atkinson* objected, saying that it was foisting the work of the section upon the general body. That it was the work of the section to consider those matters without wasting the time of the body.

*Dr. Crouse* said it would be frittering away time. The papers should be read first, and the whole subject made the basis of discussion.

*Dr. C. N. Pierce* thought a reading of the papers necessary, that it might be known what had been brought before the section.

*Dr. Ames*, Secretary of the Section, then read the paper presented by Dr. Truman, which was a detailed description of the principles involved in, and advantages derived by the use of the New Mode Heater, the Seabury Vulcanizer, etc.

*Dr. Haskell* then read his paper, describing some much needed improvements in the styles of porcelain teeth, to give them individuality of color, shape, style, size, etc.—it being very difficult to match the natural teeth when partial plates are required. Many of the teeth now made are altogether too small, being really infant's sizes, while many also are too large, horse teeth in fact. Greater variety is needed too in the shape and arrangement of the pins An improvement was needed in the quality and thickness of the enamel, as the teeth now made cannot be ground without giving them a dingy soiled appearance.

He recommended that a committee of three be appointed to confer with the leading manufacturers on this subject.

*Dr. Dorrance* read a brief paper, giving the tabulated results of continued observations of the effects of wearing plates of the vegetable bases, rubber and celluloid.

Leaving out the results of injuries caused by uncleanliness or mechanical injury from badly fitting plates, he found by his continued observations that the percentage had increased to fifty-five per cent. His treatment was the substitution of metal plates, the improvement being radical.

Dr. Dorrance read another short paper on the effect of different metals in alloys. He gave the formula of an alloy of chemically pure metals, silver 1 part, zinc 2 parts, and copper 3 parts. This makes a remarkably white alloy, and is used in the proportion of one part of alloy to 2, 3, or 4 parts of scrap gold, fusing the gold first. The solder will follow the color of the gold used, whether red or yellow.

The report of the section, including the papers, was opened for discussion.

*Dr. C. N. Pierce* inquired to what the injuries caused by the plates were attributed?

*Dr. Dorrance* said he had not discussed that point in the present paper as it was a continuation of a previous report which had covered that point. He attributed the diseased condition of the mucous membrane to the retention of animal heat, which was not allowed to escape by the non-conducting material. It was not due to uncleanliness or bad fit—one of the worst cases he had known was a patient who was extremely careful as to cleanliness. There was uniform improvement by substituting metal plates which permit the conduction of heat. He thought the coloring matter might be left entirely out of consideration, as very few plates were made of so poor a rubber as to cause the trouble.

*Dr. Pierce* said what he wanted to get at was the cause of the different results from black and red rubber.

*Dr. Dorrance* said he had never noticed such a difference. He had substituted black rubber for red without any benefit, while the substitution of metal effected a complete cure.

*Dr. Friedrichs* (New Orleans) said that at the rate of fifty-five per cent. named, fifty out of every one hundred people wearing red rubber plates must have sore mouths, in which case there were ten million sore mouths in the country. If this were the case the material would not be tolerated, which shows the folly of such conclusions. A man wore a gold plate and had hemorrhage two hours after—of course the gold plate produced the hemorrhage! If such effects were produced, it would be patent to every one in the profession.

*Dr. Ottofy* (Chicago) said that the recent investigations of Prof. Black were very satisfactory, showing that it made no difference what material was worn—there were micrococci found under all alike.

*Dr. Cushing* said that was a misrepresentation of Dr. Black. He said that micrococci were found under all *unclean* plates; when equally clean, all were alike, though close fitting rubber and celluloid plates afforded the best hiding places.

*Dr. Morgan* said that this section had insisted at the last meeting, upon being afforded opportunity for discussion, but that all that had been said to-night had been a re-hash of what has been said for years. It was a mere inference that the mouth eliminates heat, or that the natural heat of the mouth could be destructive to its tissues. The natural heat eliminated by the body cannot prove destructive to the body when in health. He had hoped that the section, having had a whole year for preparation, would have presented something worthy of consideration.

*Dr. Truman* said that for a number of years mechanical dentistry had been almost a lost art, and was now in a transition state. He feared that the advocates of bridgework were going too far. They did not stop to consider the future results of those permanent bands upon the teeth, which infringing upon the pericementum were driven home, as if upon an anvil, violating physiological law, inducing pathological conditions which they might not be able to overcome. They were going too fast and too far—daily doing work which was liable to result in permanent injury, loosening and destroying teeth, and torturing the tender peridental membrane. The principles involved should be thoroughly ventilated by the Association.

*Dr. Atkinson* said that he was glad all over to hear a man speak of *principles*, when however bold statements were made it was essential to have a strong foundation of facts. It was a mistake to say that the pericementum was irritable and tender. If there was one tissue that would bear abuse it was connective tissue.

The whole discussion was based on a lack of knowledge of principles, crowns and bridgework were inserted without any knowledge of how much loss there was at the end of the root, or how much softening around the root. We must hew to the line, even if the chips do fly in our faces. We must get out of the slough of despond; when we make a diagnosis, be sure it is not an ig-nosis. We do not know the negative, but we should hold to the positive which we do know. Bridgework, when properly constructed and inserted, is the *ne plus ultra* of replacement. I am always willing to give up my best thought, or what was the best in the category at that time, for a better. I have five times outgrown what I honestly believed to be my best thought.

The Committee on Credentials here reported that examination of the books had corroborated the statements of Dr. Metcalf.

That no copies of the transactions had ever been sent to him, which would have been done had he been a permanent member. That, consequently, he owed nothing to the Association, but was entitled to come in as delegate, and elect whether or not he would become a permanent member of the Association.

The report was accepted.

*Dr. Rehwinkel* announced that the Central German Association of Dentists was now in session, and moved that a telegram of fraternal greeting be sent, signed by the officers of the Association.

Adopted.

*Dr. Geo. H Cushing* read a Communication from the Librarian of the Patent Office, Chancery Buildings, London, written at the request of a number of readers, requesting copies of the Proceedings of the Association, for the benefit of some 200 daily readers—largely men especially instructed in scientific patents and inventions.

On motion of Dr. Taft, the Secretary was instructed to comply with the request, by forwarding as complete a set of transactions as possible.

On motion, adjourned to 9 A. M.

SECOND DAY—FIRST SESSION.

Called to order at 9:15 A. M. The President in the chair.

The minutes of the last session were read and approved.

The Committee on Credentials reported delegates present from the following organizations:

Illinois State Dental Society, California Odontological Society, Dental Department of the University of California, Chicago Dental Society, Michigan State Dental Association, Mississippi Valley Dental Society, Odontological Society of Pennsylvania, Sixth District Dental Society of New York, Chicago Dental Club, Connecticut Valley Dental Association, First District Dental Society of New York, Louisiana State Dental Society, Brooklyn Dental Society, Eighth District Dental Society of New York, Minnesota Hospital Dental College, Georgia State Dental Society, Odontological Society of Western Pennsylvania, Pennsylvania State Dental Society, Indiana State Dental Association, State

University of Iowa Dental Department, Northwestern Dental Association, New Orleans Odontological Society, Pittsburg Dental Association.

Dr. Boedecker, of New York, then introduced to the Association the celebrated Dr. Wilhelm Herbst, of Germany, who was welcomed with hearty applause. This representative of German dentistry is visiting this country for the purpose of giving to the profession his recently perfected method of filling teeth with gold by a system of rotary pressure, rubbing the gold against the walls of the cavity instead of hammering it in with the mallet, or by the old system of hand pressure. This system, which is known as the "Herbst Method," is the utilization of a new principle in operative dentistry, and has been experimentally used in this country for the last two years, but has never been thoroughly understood or appreciated until the present demonstration of Prof. Herbst himself. He calls this system his "baby," and says "I have brought my baby to this country to show it to you; if you don't like it I will drown it."

*Dr. William H. Atkinson* moved that Dr. Herbst be made an honorary member of the Association. The motion was carried by acclamation.

*Dr. Herbst* speaking in German, which was translated phrase by phrase by Dr. Boedecker, briefly expressed his appreciation of the hearty welcome tendered him, which he said, he was too much moved to express in words.

*Dr. Crouse* of Chicago then introduced Dr. Bryant, of Switzerland and several members of the profession from Canada, to all of whom the privileges of debate were tendered.

*Dr. Watkins*, of Mont Clair, N. J., offered a vote of thanks to Prof. Boedecker, to whose efforts the profession owes the visit to this country of Dr. Herbst. The President in putting the motion said that any man who could come from abroad to teach Americans one point in dentistry was especially welcome.

The report of section 4, on operative dentistry, was next taken up in order that Dr. Herbst might lay before the Association his method. The report, as read by the chairman, Dr. Darby, embodies a review of the progress made in operative dentistry in the last quarter of a century in the varieties of foil manufactured, as cohesive, Wolrab's velvet foil, etc. Also sponge gold, crystal gold, platinum, and iridium gold, etc.; in the more general use amalgam and the plastics by the best operators; in the use of rubber dam, the dental engine, mechanical and electric.

They directed attention to the band matrix of Dr. T. W. Brophy, to the diamond drill of Dr. Fernandez; to the matrix of Dr. Swasey, which also serves as dam-holder. The section having had no papers presented, asked for the discussion of these subjects.

The question as to the proper place of crown and bridgework was discussed at some length by Drs. Kingsley, Melotte, Morgan, McKellops, Parmley Brown, until Dr. Taft rose to a point of order, the subject of nomenclature not being before the body.

The general decision appeared to be that it belonged to both operative and mechanical dentistry, the treatment of the roots, and the preparation of the mouth being purely therapeutical while the construction of crowns or bridgework was as decidedly a branch of mechanical art.

Dr. Herbst was now invited to address the Association, which he did in German, Dr. Boedecker again acting as his interpreter. He described fully his methods of work, the principles on which it is based, and its superior advantages in the points of weight of gold inserted, close adaptation to the walls of the cavity, the perfect union of the different layers of gold, and the great saving of time required for the operation. The superiority of his method is seen by filling glass tubes and immersing them in aniline or carmine solutions, when not the slightest percolation will take place.

Another great improvement is found in the experience of the patient, which is much less painful or even disagreeable than under the blows of the mallet, though Dr. Boedecker advised all beginners to use the mallet for the finishing layers of gold. Dr. Atkinson said that the work of Dr. Herbst was incomplete until he had given them his *obtunder*, which is a perfect success.

*Prof. Taft* said that he had thought the formula rather startling, but after testing it thoroughly and daily for two months, he had found it a success in every instance, and that he considered it perfectly safe and reliable. The formula is simply without weight or measure, a saturated solution of sulphuric acid and crystals of cocaine, to which is added sulphuric ether until supersaturated. This is most safely mixed in a long test tube, stirred with a glass rod. If shaken in a bottle it is liable to burst the vial, or drive the cork out, perhaps throwing the sulphuric acid in the face and eyes. It is taken up on cotton and applied to sensitive dentine. The effect is perfect, but it only obtunds one layer, and must be renewed.

Dr. Taft said that for his own satisfaction he had weighed and measured accurately and found that 1 drachm of sulphuric acid would take up 30 grains of crystals of cocaine. If the latter is pure, the solution will be clear, if it turns dark there are carbonaceous particles, etc., in the cocaine.

As to the mode of action, the sulphuric acid acts as a caustic on the living tissues, the cocaine obtunding sensitiveness during the action of the acid. The sulphuric acid dissolves a layer of dentine forming a white powder of sulphate of lime, to which there is no objection in leaving in the cavity, if it is properly formed.

*Dr. Jas. Truman* thought the general introduction of this obtunder was liable to produce bad results. Using an escharotic on the dentine, which contained prolongations of the pulp tissue, would destroy that tissue to that extent, and the irritation would eventually reach the body of the pulp. The effects of sulphuric acid would be far worse even than oxy-chloride. He was surprised to see Prof. Taft endorse such a formula, and he wished to give warning against the introduction or indiscriminate use of anything so dangerous, and which must destroy the pulp tissue.

*Dr. J. S. Marshall* said that possibly some one might like to hear about the Herbst method from the standpoint of a patient. He had been a patient of Herbst's, and he would say that he infinitely preferred it to the mallet; the suffering was less than one-half. He had plugged a crown cavity of a molar, and a compound distal and grinding surface of a bicuspid. The pressure was very heavy, and his jaws so tired from the pressure that he had to support his chin with his hand. The pressing and drawing motion gave a peculiar sensation, but which was not painful.

*Dr. Atkinson* said that when Dr. Boedecker wrote him from Bremen about the new method, he thought he did not need anything but the mallet, but now, after having witnessed sixteen clinics, four of which he had seen all through—and one in his own mouth, so that he knew both ends of the instruments—he could say that he had had *a new revelation;* there was nothing to equal it for surface fillings; he had tested it and it was the hardest gold he had ever seen packed by any method. He even had him try 120 rolled gold, and iridium and platinum gold for wearing edges, and he succeeded even with that. He was ready to cry *mirabile dictu!* He had feared that rapid rotary motion of the engine would eliminate heat, but it was not so.

*Dr. Marshall* said that in his own case, in the first filling he had not experienced any sensation of heat. In the second, from some cause, at one point the gold lost its cohesion, and Dr. Herbst purposely rotated the instrument with sufficient rapidity to heat the filling and restore cohesion.

*Dr. Boedecker* said that until Dr. Herbst made this voyage, he had never experimented with the heavier foils, but he had induced him to try No. 10, and then No. 30, and he found it gave the very best results—he uses hand-pressure for placing the gold in position, condensing with agates in the engine, using garnet for the last layer, rubbing with the side of the instrument. He makes a surface so hard that ordinary shellac and corundum stone fails to take hold.

*Dr. Rehwinkel, Dr. Pierce, Dr. Waling,* and others bore testimony to advantages of the Herbst method.

*Dr. E. A. Bogue* said he would like, from the depths of his own ignorance, to ask what the "Herbst Method" *per se* is? That the *principles* involved had not been touched upon, for he had not yet found out.

*Dr. A. O. Hunt* said that those of them who were far away from the seaports had not had it demostrated to them.

*Dr. Boedecker* and *Dr. Rehwinkel* explained the process again, but *Dr. Bogue* said that all he had heard only carried him back twenty-five or thirty years, going through again with an old process which had been laid aside years ago. He did not think a single new principle had been evolved.

*Dr. Fillebrown* thought the whole thing was a question of mallet *versus* Herbst, as if no intermediate method had ever been used. He had used the mallet for twelve years, but found that force was not necessary; as hard a surface could be obtained by light hand pressure, surfaces which could resist the force of mastication and the inroads of fluids—stand the tests of aniline dyes, etc. He did not need a mallet, nor a matrix, nor the Herbst method; he could make as good operations in less time without them. It was not necessary to beat the life out of it to bring the gold into contact with the walls of the cavity and produce a good surface.

*Dr. Taft* said remarks had been made about his endorsement of Dr. Herbst's obtunder. He had only spoken of his own experience as to its action; that Herbst had used it for five years without any unfavorable results; it succeeded beyond anything else. He said that the reasoning of Prof. Truman was hardly born out; the effects were not so violent as his language would lead to believe. Sulphuric acid had been used for years in alveolar abscess. If its action was so violent it would be totally impracticable for the purpose named. It is an escharotic, but its effects are limited to the surface; it affects only one layer; it is not an agent capable of doing the mischief imputed to it by Prof. Truman. He only feared some would refuse to use an agent which might be of great service.

*Dr. Rehwinkel* said that *veratria* was a valuable obtunder, but a very dangerous one—the formula for its use is one-half grain veratria in three drops absolute alcohol, to which is added tannin to saturation, when ten drops of glycerine is added. It is applied after the dam is in place, and the cavity dry. It should be borne in mind that it is exceedingly poisonous, outside of the tooth, but is a fine obtunder.

*Prof. Truman* said that Dr. Taft had shirked the whole question. That it was treating a very delicate tissue; an escharotic could not be used on the dentine without disturbing the main body of the pulp. The effect of a remedy in alveolar abscess, or in pyorrhea alveolaris had no connection with the question. He would add that Herbst had used it in Germany where the teeth were of very different texture, being very dense; here we have teeth of very soft structure. That it could not be used with safety here.

*Dr. Boedecker* said that he wished to correct a misapprehension. Last year, in Bremen, the obtundent being mentioned, Dr. Herbst had taken him to see a patient—a child of very delicate constitution, ten or twelve years of age, with teeth in which he himself would not under any consideration, have put gold. He asked Herbst why in the world he used gold in such teeth. Dr. Herbst replied that after being treated with his obtunder, they would stand gold better than anything else. He examined them critically, and tested with instruments. He found the edges absolutely perfect. He showed him three such cases and all were standing well, without any bad results whatever. In one case the nerve had been exposed, but the pulp survived the treatment of the dentine with this obtundent. He had tested it with ice water, etc., and it was as alive as ever. He had had it used in four of his own teeth, and hoped to show them in after years.

*Dr. Morgan* said that he was not chemist enough to say what new compound was formed, but he knew that some remedies were self-limiting, and if this much was known their use was not empirical. It makes no difference how strong or how poisonous they were; if we knew the exact limit of their action they were perfectly safe to that extent. But it would not do to grope in the dark. Take aqua-ammonia; its action was very superficial; but on the other hand take arsenic—we don't know when its action will cease; it may run on for months or even years. We want to know how an agent acts on living tissue, and whether it satisfies itself with limited action.

*Prof. Brophy* said that he had used various substances as obtundents, and found that it was never safe to use an escharotic except at the risk of impairing the vitality of the pulp. We have all seen pulps devitalized by obtundents.

*Dr. Sitherwood* said that as he understood it, those who had tested it, and knew its chemical action, had demonstrated that it would not do any harm.

*Dr. Taft* said that it had been fully demonstrated.

It was composed of sulphuric acid, hydrochlorate of cocaine, and sulphuric ether; that it acts on the calcareous portion of the dentine, dissolving a layer and forming the sulphate of lime. It cannot go beyond that point; it is satisfied with what it has dissolved.

*Dr. H. A. Smith* said that the chloride of zinc being largely used as an obtundent, he thought possibly that cocaine might unite with it, as with the sulphuric acid, and prevent the pain caused by its application. As an escharotic it acts only to a defined depth, but it might cause inflammation and hurt the pulp.

*Dr. Atkinson* said it was desirable when speaking of any agent, when we do not positively know, not to assume knowledge. We do not know what the combination is; even chemists are not satisfied—they are at loose ends. It is dealing empirically, but if it is successful and does no harm there is no objection to its use. We should not be scared out of our boots by old fogies who don't know the first letter of the alphabet of chemistry. Is chloride of zinc self-limiting? It sets the pulp at ease by transforming it to the hypo-chloride of albumen—as clear as glass to the apex. An agent, when self-limiting forms a compound which is not soluble in the agent. But chloride of zinc is not that sort; neither is arsenic; we don't know where the mischief will end—we put in arsenic, and it don't hurt—"all quiet along the Potomac" until McClellan left!

I wish to defend the man who can't speak English, and I wish I could speak German to tell him how I love and admire him; not as a personality, but for his principles; I praise no man, except as he is the representative of the world of light and intelligence which gives us all we have. They say I fight and quarrel! No; I love the man who reflects the truth. I repudiate the term

*ours*; it is not mine or thine. We must put ourselves in the line of reception, and let our minds be convinced. Then we wouldn't get mad and say: "I'll squeeze you!" I don't care, I am either above or below criticism. I love the truth, and in no body of men is it found as among dentists; they are the embodiment of light and truth—the sons and daughters of the Almighty.

On motion, adjourned to 8 P. M.

### SECOND DAY—SECOND SESSION.

Called to order at 8:15 P. M. The President in the chair.

The minutes of the morning session were read and approved.

The discussion of Sec. IV., Operative Dentistry, was continued.

*Dr. Darby* moved the consideration of the Appliances mentioned in the report of the committee, no papers having been presented.

There was a little tool devised by Dr. Fernandez for dressing corundum wheels and points, consisting of a flake of black diamond set in a steel instrument. The wheel being revolved is shaped and dressed as wood is turned in a lathe. It can also be used in dressing fillings, and will be found a handy little tool to have in the operating case.

*Dr. Brophy* illustrated on the blackboard his matrix, made from a thin band of steel or ribbon similar to the fine saws used for dressing fillings, and retained around the tooth by means of a screw. This matrix yields sufficiently to allow of carrying the gold with wedge-shaped pluggers, closer to the borders of the cavity than by any other method, the first pieces being introduced between the band and the tooth, making a firm margin at the cervical border.

This is similar to the band-matrix of Dr. Herbst, except that the latter is soldered, and being constricted around the neck, and standing away from the cervical wall, requires a wedge. Another advantage of the Brophy band-matrix is that the gold is all condensed in the direction of the roots of the tooth, in horizontal layers, with no diagonal layers with a little corner to flake off.

The filling should be finished with metallic strips. This matrix is especially valuable for cavities on the posterior surface when the posterior tooth is missing, instead of the lump of shellac which Herbst uses in such cases. This band-matrix can be placed where even floss silk will not pass. The screw is tightened either with a watch key or a little wrench especially adapted.

On the inferior teeth, the screw should be adapted on the lingual surface, which is more likely to be straight and afford firm hold.

*Dr. S. E. Perry* exhibited and described a hand piece attachment which combines the advantages of the Elliot Suspension and the Bonwill with the Hodge hand-piece, by means of pulleys, etc., giving a steady lathe-like motion. A spiral spring supporting the arm relieves the hand from all weight. A braided freting-line is used, put together without splicing, avoiding the "jump" of the overlap of a corset cord. By the use of rubber bands on the engine wheels and on the spindle pulley, the line can be used with loose tension without slipping, while the right angle attachment being secured directly to the pulley-block, great steadiness of motion is secured. All the parts can be readily taken apart for oiling and cleaning.

There is also a shield to protect the hand from the main pulley, and side-bearings to the small pulleys, giving a free, light feeling to the hand piece.

*Dr. S. C. Watkins* (Mount Clair, N. Y.,) exhibited six instruments—three *double enders*—designed for finishing amalgam fillings in approximal cavities (which in the majority of fillings *put in by others* project beyond the margin). They are spring-tempered, with knife-edge, and can be used in any position, drawing in either direction, reaching the most difficult cavities. With a delicate touch and very little strain, all excess is removed.

He also exhibited his pattern for tooth brush, which is narrow at the end, and designed to reach the posterior, and lingual, and labial surfaces of all the teeth. Apparently quite similar in design to the Prophylactic tooth brush of Dr. Rhein, manufactured by the Florence Company.

*Dr. Guilford* exhibited, and illustrated on the blackboard a matrix, held in position by a bar crossing the crown of the tooth, with ears dipping down to which the band is secured by small screws—avoiding the necessity of forcing the teeth apart, the band going only half way around. The different bands have curved dips in different positions to meet different sizes and shapes of approximal cavities, and can be bent with pliers to fit closely to the neck of the tooth. With this matrix, when the cavity does not approach the masticating surface, it can be cut out from the side, a slit being cut in the band and laid back, forming a perfect pocket, with no sharp edge for the instrument to catch against.

Section IV. was passed without further discussion.

Sections II. and III. not ready to report.

*Dr. Frank Abbott*, chairman of Section V., reported that though little that was interesting in the line of work of this section had been published during the past year, much work of very great importance was being pushed forward which would, in due time, be given to the profession.

Dr. Abbott reminded the Association that, in 1879, he had published the result of observations from which he attributed caries of human teeth to a process of inflammation. This view was then opposed, on the ground that there was not sufficient living tissue in the dentine to permit of inflammation, even from irritation of caries. He said that the more he had studied specimens of carious teeth under the microscope, the more firmly he became convinced that his original position was correct. More recent investigations by Drs. Boedecker and Heitzman (published in the Indiana *Practitioner*) fully confirm the views which he had set forth seven years ago showing the existence of inflammation of the dentine when only slight caries, or even no caries at all, is present.

Nothing of importance in Anatomy or Histology had been brought to the notice of the section, and only one paper offered. This he proceeded to read.

The paper was illustrated by magnificent drawings by Dr. Heitzman, entitled: Hyperostosis of Roots of Teeth.

Under this single term he included all the forms of pathological new growths of cementum, known as Osteoma, Exostosis, Hypertrophy of the Cement, etc.

He enumerated as causes of this disease, irritation of the cementum, either through caries of the crown or neck, or through exposure of the pulp; localized irritation of the pericementum; irritation of the pericementum of the upper teeth from gravitation after loss of antagonizing teeth; and the irritation from outgrowths of cementum.

He said that *tumors* had been considered as the result of chronic irritation of the mother tissue, caused by local irritation, Cohnheim claiming that they were the result of a "misplacement of embryonal germs." Chronic irritation of the cementum might result in a new formation, but the question for consideration was--can it occur after the cementum has been fully formed? This question he was led to answer in the negative from microscopical studies of the tissue formation, which he concluded was, in most instances, a fœtal malformation.

If the roots of a carious tooth were found in a hyperplastic condition, it would be inferred at once that the inflamed pulp had led to pericementitis, and this to hyperostosis of the roots, but if all the roots were uniformly enlarged, or fused together it would be just as possible that this condition had existed before caries made its appearance.

From a careful study of a very large number of specimens he felt convinced that the teeth had been sound, and the pulps alive when the bony growth was formed, the growth ceasing with the life of the pulp. When a tooth is extracted for the relief of pericementitis and found exostosed, it would scarcely be supposed that the pericementitis had caused the exostosis, but rather the contrary, the enlargement being the primary, and the pericementitis the secondary features.

Whether cartilage or fibrous connective tissue is formed first, the lacunæ contain living protoplasm, and the canaliculi hold for tenants fibers of living matter, only one portion of the protoplasm being transformed into basis-substance, forming the matrix into which is infiltrated the lime-salts. Augmentation of the cementum is impossible without a preceding augmentation of of the medullary tissue, this being caused by increased nutrition or irritation.

Boedecker has demonstrated that in normal cement the whole basis-substance is traversed by a delicate reticulum containing threads of living matter, even pathological cementum being endowed with the same properties of life. The process of the development, growth, enlargement, destruction, and re-formation of cementum can be understood only as it is admitted that it is a living tissue. The diagnosis of hyperostosis is extremely obscure, the pain usually not being localized; several teeth being frequently extracted before relief is obtained.

The different varieties of hyperostosis he divided into four heads:

1. Circumscribed hyperostosis.
2. Diffused hyperostosis, with roots separated.
3. Diffused hyperostosis, with roots united.
4. Two or more teeth united through hyperostosis.

These four groups were again subdivided:

1. (*a*) Osteoma on body of the root.

(*b*) Osteoma on the apex of the root—varying in size from microscopic to the size of a lentil or a pea, and usually with nodular surface, and appearing on teeth of which the crowns have been more or less destroyed by caries, with probably long exposed pulp, the death of the pulp stopping the formation, which must have commenced long before the exposure of the pulp.

In (2) the roots are affected without any symptom of disease upon the exposed portion of the tooth, the disease probably dating from the beginning of the formation of the cementum.

In these the hyperostosis invades the roots from the apex to the middle, or two-thirds, or even the whole length of the roots, sometimes even overlapping the enamel, the roots appearing clumsy and shortened with the mass partially filling the space between them.

In (3) the apices may be (*a*) free and straight, or (*b*) free and curved, or (*c*) the roots may be united their entire length.

(*c*) is quite common, this being almost the normal condition of wisdom teeth, only slight furrows indicating any separation of the roots.

(4) is of rather rare occurrence, and it is probable that at least one of the germs was malposed at the time of embryonic arrangement, and that the alveolar septum did not form at all.

One of the collections of teeth from which these studies were made forms an exception to the rule that only bicuspids and molars are subject to exostosis, in the specimen referred to an upper canine being completely united to a lateral incisor having a common apex with a single large foramen. The very fine illustrations of microscopic studies of these formations showed the structure of the osteo-dentine, and the vaso-dentine of Wedl; the hyperplastic cementum traversed by medullary canals carrying central blood-vessels, the canals and blood-vessels being unquestionably in communication with the blood-vessels of the pericementum, hyperplastic as well as normal cementum being a living tissue throughout, between the basis-substance and the filaments of living matter a slow circulation going on, carrying nourishment to and taking away effete material. By these studies and illustrations the pathological changes of a pathological tissue become intelligible.

It being after 10 P. M., adjourned without discussion.

THURSDAY, August 5.

THIRD DAY—FIRST SESSION.

Called to order at 9:30 A. M., the President in the chair.

Minutes of the last meeting read and approved.

*Dr. C. Cahoon* asked consent to present a supplementary report from Section I.

The chair stated that this could only be done by unanimous consent.

*Dr. Marshall* thought it should come in regular order. That other sections were ready to report.

Assent being given, Dr. Ames read a supplementary report describing Dr. J. R. Knapp's gold crowns with porcelain faces; also his blow-pipe which uses compressed nitrous-oxide gas in combination with illuminating gas, forming a compound oxy-hydrogen plow pipe.

*Dr. J. R. Knapp* being called on said that he could say nothing beyond what he had exhibited in his clinics. He would be pleased to show his inventions and give any information desired.

*Drs. Kingsley* and *Abbott* commended the work very highly.

Section I. passed again.

The Committee on Credentials reported delegates present from the Vicksburg Dental Association; Wisconsin State Dental Association; Fifth District N. Y. Society; Alumni of Dental Department University of Pennsylvania; Northern Ohio Dental Society; Susquehanna Valley Dental Society; Kentucky State Dental Society; New York State Dental Society; Missouri Dental College.

*Dr. Wm. H. Atkinson* said that he did not hear the name of the Society represented by Dr. J. R. Walker, of New Orleans.

He understood that that gentleman had presented his credentials, which had been accepted, and his dues paid; that his dues had been returned to him on the ground that charges were pending against him from some local Society. By the laws of the United States every man was entitled to be considered innocent until proved guilty. That it was a small business for this Association to espouse local quarrels. Dr. Walker was known as an efficient worker. He remembered him in the very infancy of its organization in 1859. On one oocasion he himself was not received, not being accredited from any local organization. This was the pet method of local organizations of dudes and cranks. At Washington they had made him the first President, to heal the wound. This Association was not the creature of local societies; it had the right to receive members who were worthy, irrespective of the petty malice of local organizations.

*Dr. Crouse* said that the committee was competent to reject on information received, and that the only way to keep right and square was to work up to the laws of the Constitution.

*Dr. Atkinson* said he did not ask them to violate any law.

The report was accepted.

*Dr. Pierce* reported that the Committee on Voluntary Essays had received two papers. One had been returned as incomplete; the other, containing seventeen printed pages was too long to be read as a whole. The committee recommended that certain portions be referred to the Publication Committee.

*Prof. Taft* thought that if the paper was a good one the Association should hear it. That persons who devoted time and labor to the preparation of papers, containing information profitable to this body, were discouraged from further labor if they were thus cast aside.

*Dr. Kingsley* rose to ask for information. He wished to know whether papers in the hands of that committee passed out of the control of the author?

The chair replied that they did; they became the property of the Society.

*Dr. Kingsley* thought that in that case it was not fair to mutilate them. They should either be accepted or rejected as a whole. In the latter case the paper should be given to the author, who might take it elsewhere.

*Dr. Atkinson* said that the organic law of the Society entitled the committee to do what they thought proper. If they did not have the brains and heart to do what was clean there was no remedy, but it could not be expected to do a-b-c-d-arian work, nor to publish drivel *ad nauseam.*

*Dr. Allport* said that committee was appointed for the purpose of determining what papers were proper to be read before the body, and it was fair to suppose they would do this with judgment.

If the committee could not stand the whole of a paper certainly the whole body did not want to read it. The paper should go to the Publishing Committee, who would use what they saw fit.

*Dr. Geo. H. Cushing* said the Committee on Voluntary Essays should not delegate their work to the Publication Committee, which had work enough of their own to perform.

*Dr Thompson* thought it was the duty of the Publication Committee to decide whether a paper should be published in full or in abstract.

The report of the committee was adopted.

On motion of Dr. L. C. Ingersoll, it was made a standing resolution (the Association having no by-laws) that at every meeting of the Association, it should be the duty of the Executive Committee to provide a register, wherein all members, and all visitors to the hall should register their names, residence, and hotel.

The discussion of Section V. being the order of the day--

*Dr. Atkinson* said this was the only section in which clean work could be done. He had hoped more papers would have been presented.

He spoke of the different terms used, as solidal pathology, humeral or fluid pathology, cellular pathology, and pragmatic pathology, causes and consequences, and the effect of unseen elements. He said we should throw away all irrelevant matter and endeavor to comprehend how changes take place. He considered the term hyperostosis doubtful nomenclature; it was rather hypercementosis. It was trying to get at the work effected by the several plans or modes of arrest of radiance that operate all things in pathology. We can't see a molecule—no power of the microscope can reveal a molecule; it is an ideal body, as is an atom. They are crutches to help the mind to grasp what it is that is held in combination till it becomes perceptible to the senses. He said that if he had time to go over the whole paper read, he would show what was worthy of attention, and what was mere fuss and feathers; then he might bless them, and be blessed himself. The illustrations were the very culmination of drawing. He said that, as a rule, they did not know enough about histology for it to be profitable for him to go into it very deeply.

Doctrines were pronounced *settled* that were mere hypothesis and assumption. The assumption that the predisposition dated from *in utero*, and that the work was carried on only during the life of the pulp, was not proven. It was potentially resident in the tooth germ, and with new energies we get increased deposits of protoplasm, with deposits of limesalts to make it visible as we see it. It is a fact that roots of teeth have ten, twelve, or fifteen distinct laminæ of cement corpuscles laid on and fusing with adjacent teeth, he having seen five in one solid phalanx. No one could say that this had occurred before their eruption in the line in which they stand with their biting surfaces in perfect apposition. The merest glimpse of law shows that want of use raises hell in the shape of gout, cancer, tubercles, etc.

Lack of knowledge of the so-called peridental membrane leads into new trouble. It is said when the periosteum is saved we may expect reproduction of bone. The periosteum is not a bone producer; that is the work of the substratum of ostoeblasts. We justify one error by another. We need to do in intellectual growth as in bodily growth—we must take in breath—take from it what we need—blow it out, and never see it again. It is the same with knowledge.

Deducations ought not to be presented to a body of men who are less endowed in these matters. If you only knew how my protoplasm and mesentery boil to see men use the ideas of other men! We want not contention but emulation. We should not ask *why*, but ask *how*. The microscope reveals *how*, but how is it often done? If a man is cut open after he is dead we find only the tracks--we don't know what has been there. As each man tries to hold his own, so do the corpuscles or molecules. Each individual body, in the effort to maintain its own existence, offends another.

We say arsenic causes certain results; it is the energy behind it that causes; the arsenic is only the vehicle.

(Time up—unanimously extended.)

Arsenic is a metal; it has no affinity for the tissues; it must be oxidized. By contact the metal is changed into a salt; a disruption of the molecule takes place and the poison comes in. Mercury follows the same law.

If we believe in poly-pharmacy we are primed with sorrows and must tramp through swamps.

Nutrition follows the same law; it is operated by the type behind it.

The committee which received my paper *Ripening and Ripeness* could not understand what I said, and wrote their own shame on the back of my report. They wanted to kick it out, but I have been thus treated so often I am not afraid!

If this is the last word I have to say, let it be:

Obey the sense of righteousness, and never violate your conscience in the least thing. Walk in the light, in the evidence of the excellence of knowledge and wisdom, as presented to this body.

*Dr Thompson* inquired if there might not be a nodular idiosyncracy tending to produce such deposits on the bones and teeth ?

*Dr. Atkinson* said he would offer a Delmonico dinner, a pair of clean sheets, and a week's lodging to any man who would give him a well authenticated instance of hypertrophy or exostosis on a tooth which had never lost its occlusion.

*Dr. Ingersoll* said this was a high-toned paper, and in the right direction; an investigation in fields which were comparatively new, and where progress is much needed. He remembered the phrase inflammation of bone twenty-five years ago. It was discarded, not being understood Then it was called sensitive dentina, but the question still remained—what makes it sensitive ? If we admit that it is due to inflammation of the dentine, it must be the result of vascular activity, and the question arises: Is there a vascular system in dentine?

Confusion arises also from a misconception of the term inflammation and what it implies. All the phases of inflammation are not manifested in every tissue—inflammation of cartilage is as genuine as inflammation of vascular tissue. It has been said that because the blood cannot enter the tubuli of the dentine, there can be no inflammation, but that does not follow; there must be circulation or there can be no nutrition; there is nutrition, therefore there must be circulation. With vascular action there may be inflammation of dentine. The dentine is *sensitive*, that is, it is in pathological condition of inflammation. An exposed, healthy, fresh pulp is free from pain until it takes on pathological conditions and becomes inflamed, then it is painful.

The old pathologists said that teeth were bones; strictly speaking, there is bone in tooth structure; there is bone in cementum, and yet cementum is not strictly identical with bone; it is modified bone tissue. Hypercementosis would be a better term than hyperostosis or excementosis to distinguish it from exostosis. He questioned whether the term hypertrophy was quite correct; he would look upon it rather as tumefaction. We may have hypertrophy and pathological irritation, or there may be physiological excitation; they are not synonymous. There is normal excitation, but not normal irritation, though the first may become the second. There may be physiological cementosis, and there may be also pathological cementosis; one is healthy, the other is abnormal and unhealthy. If we accept the theory of inflammation of dentine this becomes another factor in decay. The processes of nutrition are analagous throughout the entire body; so of the process of inflammation. The work of construction and destruction is uniform. Dentine and bone are constructed on very much the same plan, except that dentine is tubular, bone cancellated; the medullary tissue undergoes the process of inflammation; there is swelling at the expense of the walls of the cancelli, and the bone is softened; in the dentine the swelling of the dentinal fibrils is at the expense of the hard tissues. In what we call demineralized dentine, the tubuli are broken down by the expansion of the fibrils.

Thus germs and acids are not the only factors in decay, inflammation bears an important part.

*Dr Wells* said if he had understood Dr. Atkinson correctly, he had said he had never known a case of hypertrophy of cementum where the tooth had proper occlusion; that Dr. Atkinson had offered so many good things, that he could not afford to miss the chance; that he had positive proof in his own mouth, in a tooth which Dr. Fuller had extracted. The occlusion was perfect, yet the root was hypertrophied.

*Dr. Atkinson* said there were two extremes of physiological action—hyper-nutrition and marasmus. One was seen in the illustrations, the other was seen in the pointed roots of teeth of aged persons, where successive layers of cement had been taken off.

*Dr. Morgan* said he considered the paper one of great value; the drawings were the most beautiful he had ever seen. He could accept most of the statements made, but thought the inferences incorrect. He did not think it true that no increase of cementum could occur after the death of the pulp. This being the function of the membrane he thought it would continue as long as the membrane was healthy. The membrane not being in connection with the pulp, he did not see how the death of the pulp could affect the function of the membrane. He said that Dr. Ingersoll had made a clear, concise talk, but that he had assumed some points with which he must take issue. He had claimed that all sensitive dentine was the result of inflammatory conditions. He (Dr. Morgan) considered the teeth somewhat analogous to the other structures. In general surgery there was healing by first intention; in good health a wound would heal by first intention without inflammatory products, though there might be great sensitiveness from increased circulation. This might be possible in teeth; in fact, in the efforts of nature to repair loss of substance, as where the teeth were worn by attrition, the tubuli filled in with lime salts; the increased sensitiveness of the dentine might be due to the increased circulation in the efforts of nature to repair the losses due to absorption. Dr. Ingersoll had stated there could be no decay without inflammation.

*Dr. Ingersoll*—no sir; you misunderstood me—may I correct? In regard to inflammation, I said there was a peculiar sensitiveness in dentine which was normal and physiological; but that it was not painful without inflammation; a twinge of pain is the evidence of inflammation. In healing by first intention there is no inflammation. Authors are confused in the use of terms. Stricker says inflammation always includes suppuration. Another includes only redness and heat as constituting inflammation. Irritation is as truly inflammation as suppuration; it is one of the series of processes—each one being inflammation in one of its stages. Before healing we have irritation which is the first step in the inflammatory process. One man says there is *no inflammation*, meaning merely there is *no suppuration*. In healing by first intention, there is no loss of tissue. In healing by second intention there is loss of tissue and restoration of parts.

A motion was made to pass the subject, followed by calls of Abbott! Abbott!

The President stated that the reader of a paper had the right to the closing speech.

*Dr. Abbott* said that his conclusions were merely his own opinions based upon careful observations. He did not believe that such increase of cementum could take place unless all the functions of the tooth were in working order. He believed that if the pulp was dead it destroyed the function of the pericementum. The question of occlusion was a different question altogether. One gentleman says he has had positive proof on the point in his own mouth. Another takes the position there may be a condition of the system inducing such results regardless of occlusion.

*Prof. Taft* referred to a case where antagonism was perfect, and yet a large increase of cementum.

If this is true, Dr. Atkinson owes the dinner; and he will pay it! My impression is, that when an inferior antagonizing tooth is taken out, the mere force of gravitation will cause an irritation that will create an abnormal growth of tissue.

The preparation of these specimens, and the illustrations, has involved an immense amount of work,

*Dr. Morgan*—All honor to the man that did it, and to the man who had it done!

*Dr. Abbott*--In this way only can facts be ascertained.

We see here, malformations of the teeth. As regards the extra growth of cement on the roots, no one has ever said that he knew when it began. It may have been simultaneous with the growth of the roots after the formation of the crown. We can't take children's teeth out one after the other to ascertain these facts. We only get the teeth after death, or after they are removed to relieve the suffering caused by the growths already completed.

Preparation for the Association should be the work of the year. As soon as I get home, or before I get there, I begin to think what subject shall I take up, and I get to work on it within an hour after I reach home. I get my specimens ready and take them to Heitzman and we work together on them. The subject of inflammation is so big, in the tooth or anywhere else, that I must now leave it where it is, but I hope what has been done will induce some one else to take it up and find out whether I am right or wrong. I have taken one side, you can take the other. Subject passed.

Before the next section was called, the Treasurer of the Association, Dr. Geo. W. Keeley, of Oxford, Ohio, presented a few interesting statistics to the Association, among others being the following: The Association dates its existence from a preliminary meeting held at Niagara Falls in August, 1859, the organization and first session being held at Washington, D. C., in July, 1860. Up to 1884 it has had altogether 747 members, with but 146 at any one time. At present there are 270 members, 32 of whom were admitted at the present meeting. Of the 23 persons whose membership dates from 1867, seven have been honored with the Presidency of the Association. They are: Dr. W. H. Atkinson of New York; Dr. J. Taft, of Cincinnati; Dr. G. W. Keeley, of Oxford, Ohio; Dr. W. H. Morgan, of Nashville, Tennessee; Dr. G. H. Cushing, of Chicago; Dr. H. J. McKellops, of St. Louis, and Dr. J. N. Crouse, of Chicago.

*Dr. Marshall* read the report of Section VI. There had been presented the description of the contents of an ovarian tumor, consisting of a partially developed jaw with teeth, and a mass of long hair; also by Dr. Hooper, of Louisville, an osteo-sarcoma, and the report of a case of exsection of the inferior dental nerve, and a paper by Dr. A. W. Harlan, entitled

BACTERIO THERAPY.

The section reported that they had not expended the sum of $200.00 assigned them for scientific investigations, but had been engaged in maturing their plans, and designed to use it during the coming year in the employment of men of recognized talent and ability to make a special study of Pyorrhea Alveolaris, in all its phases—its deposits, exudations, secretions, microbes, etc., with the characteristics of the saliva and urine, keeping careful records of their investigations.

The report was accepted.

*Dr. Hooper* read his paper, giving the details of a case of exsection of the inferior dental nerve, using dental engine fissure burs cleaned with steel file brush. Chloroform was administered, and two and a half inches cut from the nerve. The wound was dressed with horsehair drainage and healed readily, leaving scarcely any cicatrix. The patient had suffered for years with so-called facial neuralgia, and had all the teeth extracted without obtaining more than temperary relief. Since the operation there had been no recurrence of the pain, the only feeling being a slight numbness of the chin, with a tingling sensation.

Dr. Hooper also exhibited a large piece of necrosed bone removed from the left inferior maxillary of a boy twelve years of age, the result of an abscessed tooth which had been allowed to break on the outside, having been treated for nine months by a physician.

Dr. Hooper related also a case of supposed cancer, treated by several physicians and lanced several times, all arising from a dead nerve in the right central incisor, probably dating from a fall down stairs several years previous. The pulp canal was found filled with very offensive watery pus. The "cancer" was cured and also headache, hardness of hearing being much improved by proper treatment of the tooth.

The paper of Dr. A. W. Harlan on Bacterio-Therapy was next read by the author.

*Dr. Harlan* said that the proceedings of a scientific body now would not be considered complete without a dissertation on micro-organims. It is not yet clearly understood how these germs operate in producing pathological conditions, though it is claimed that an isolated microbe, cultivated in a sterilized medium, will produce, in a healthy subject, all the symptoms of the disease from whence the microbe was taken. The labors of Pasteur, the discovery of the microbes of Asiatic cholera and the bacillus tuberculosis; the existence of actino-mycosis in man as well as animals—all tend to support this theory, though whether the disease is caused by the mere presence of the germs, or by the soluble ferments excreted by them is still a question. This is not an essential question, however; we know that they are inimical to healthy living tissues, and the means of destroying them, or of neutralizing their products, is the important point, the investigation of which constitutes what may be termed bacterio-therapy.

Systematic experimentation has been undertaken to ascertain the relative value of various antiseptics and disinfectants which without such determination are used empirically. The distinction between antiseptics, disinfectants, and germicides or *microbicides* was drawn, and the conditions described, which indicate one or the other, or both.

In dental surgery choice should be made of those least objectionable to taste or smell, and least injurious to instruments and garments.

The list from which to choose is long, and the effect sought to be obtained should govern the choice. (For table of comparative values, see Proceedings Ill. State Dental Society, 1886.)

At the conclusion of the paper Dr. Marshall desired to present a supplementary report, Dr. McMillan, of Kansas City, having presented an interesting pathological specimen--the skull of a negro who died at the age of 55 years, having congenitally anchylosed jaws--or rather it was supposed to be congenital; his history known from the age of three years. He was an habitual drunkard, and took his food and whisky through the opening made by missing front teeth.

*Dr. Atkinson* inquired about the jaw-bone and long hair. He wanted to ask whether or not the hair grew directly from the bone?

*Dr. Marshall* said there was a partial development of the superior maxillary and teeth, with skin tissue and hair upon it.

*Dr. Atkinson* said he had been at a loss to comprehend how hair follicles could develop from the jaw-bone.

Subject passed.

Section VII. Physiology and Etiology. Offered in a paper from Dr. Storer Howe, of Philadelphia, on the value to the dentist of litmus-paper tests of the oral fluids. He had prepared charts for keeping a record of results which he hoped they would carry home with them and use through the year. A record of the condition of the oral fluids, whether acid, alkaline, or neutral with a record of dental lesions would be found very valuable and suggestive of remedies. This would, at least, furnish facts on which to base theories.

*Dr. A. H. Thompson*, of Topeka, Kan., read a paper on Protoplasmic Nutrition and Molecular Metamorphosis in the Dental

Tissues, in substance as follows: Although protoplasm in its primitive simplicity was not found in any animal tissue, except in the earliest embryonic stages, it was impressed with mysterious powers of differentiation into specified organs and tissues of complex organization.

Why all the forms of all the tissues should assume special shapes and characteristics and powers, conveyed by the minutest cells, is the great mystery of life which we cannot comprehend.

Each tissue selects, from the stream of nutrient pabulum, the particular elements necessary to elaborate itself, whether nerve, or muscle, bone, or tooth enamel.

Nutriment is carried in, and waste is carried out, each tissue performing its allotted work in the economy of the organism.

It is a mooted question whether the enamel should be classified as a vital or as an exfoliated product, being an epithelial, though calcified, product.

We cannot believe that it is totally devoid of life, nor yet that it is vital to its periphery. The organic areas of protoplasmic elements which exist in it while the life of the pulp is maintained, prove it to be vital, but when the pulp perishes the enamel is as dead as hair or hoof cut off from nourishing currents.

The pulp selects from the pabulum the organic and inorganic elements required to construct the different tissues.

Dentine is formed in a protoplasmic matrix, by the reduction of supplied lime salts formed in successive capsules, pierced by fibrils around which permanent tubuli are formed. The contents of the tubuli, the fibrillæ, convey nutrition into and waste from its tissue.

The enamel is formed in an analogous manner, differing only in detail. There are areas of living matter throughout the enamel also, perhaps a reticulum of protoplasm penetrating the substance of the enamel prisms, conveying limited nutrition with corresponding elimination of waste to some extent at least by means of the connection with the living matter of the dentine at its periphery. This movement is maintained by osmosis, upon which depends continued life in all animal organisms—man himself being but a modified sponge, straining out nutritive elements and casting back waste products into the ever-flowing stream. The hunger of the tissue causes it to draw in the pabulum, the more vital and vascular the tissues the greater being the absorption of pabulum, and the greater the consequent throwing off of waste. The more dense the structure the less the nutrition and waste.

The dental tissues must be nourished by this power of osmosis through the fibrillæ of the dentinal tubuli, which anastomose with the canaliculi of the cementum, the circulation being thus continuous through the two tissues, and maintaining some vitality in the dentine even after the death of its life-source—the pulp. By similar anastomosis between the dentinal tubuli and the areas of living matter in the enamel, nutrition is also conveyed to the latter tissue, though in a minute and almost unappreciable degree.

Molecular metamophosis is therefore possible throughout the entire tooth structure, and its tissues subject to the pathological conditions of the circulating fluids. Increased density or calcification in tooth structure from eruption to adolescence and old age being admitted and recognized, decalcification must also be admitted. This is, indeed, recognized as the result of the accelerated physiological activity of gestation and lactation, a molecular breaking-down occurring, the resulting material being appropriated by the growing fœtus or child.

In other conditions of health (or ill-health) we see similar results, teeth that were of good structure for years taking on a condition of softening, something acting through the circulating fluids disintegrating and carrying off the lime-salts, which have, perhaps, a destiny as food to other parts of the system, or are, perhaps, only carried off merely as waste products. We do not yet understand the mystery of molecular metamorphosis constructive and destructive. The bones lose their phosphate of lime, and the brain loses its phosphorous, until a phosphorous famine begins all over the body. In some diseases molecular construction is held in abeyance while waste still goes on; in others construction goes on while waste is loosened, with consequent increase to the density of the tissues.

This we can observe in the dentine, and probably takes place in the enamel also.

If removal of waste is not followed by compensating reconstruction softening of the tissues will result with loss of resistance to caries.

In certain inflammatory conditions intense molecular activity takes place with breaking down and removal of elements followed by molecular synthesis, the basis substance as well as the calcific elements being reproduced, a molecular work of which the mystery is as yet impenetrable.

After the reading of this valuable paper, the Committee on Credentials offered a supplementary report, delegates being present from the First District Dental Society, New York; Wisconsin Dental Society, and Minnesota Dental Society.

The Auditing Committee reported favorably on the accounts of the Treasurer and the Publication Committee.

The President called attention to the display of rare drugs made at his solicitation by Sargent & Co., of Chicago.

Adjourned to 8 P. M.

THURSDAY, AUG. 5th—THIRD DAY—SECOND SESSION.

Called to order at 8:15 P. M.

After reading of the minutes of the morning session, an amendment to Section 1 of Article VI. was offered by Dr. Marshall, taking Anatomy from Section V. and creating Section VIII. to be called Anatomy and Surgery.

*Dr. Atkinson* moved to amend by fusing Sections II. and III. as Section III, making the new Section II.

*Dr. Thompson* thought if anything was done in this direction it should be overhauled; morphology should be introduced.

Further consideration of this subject postponed.

Section VII. continued.

*Dr. H. A. Smith* read a paper entitled

THE TONGUE IN HEALTH AND DISEASE.

*Dr. Smith* said that the section had recommended the consideration of this subject, but that he had labored under great difficulty from lack of reliable data, although the condition of the tongue had always been regarded as affording an index to systemic conditions.

Being conspicuously in view during the dental operations, if the varying appearances presented were fully understood by the dentist it might be made of benefit to the patient.

The teeth, if healthy and intact, form a protecting wall around the tongue, but if diseased or broken down, they become the source of a variety of diseases of that organ; on the other hand, when the tongue is the seat of organic disease, it becomes an offence to the teeth.

Vegetable parasites form the first great source of diseases of the mouth and tongue; this the source of *thrush*, a membranous disease of the tongue and the inside of the mouth, to which children fed with a spoon are peculiarly liable, not having the neutralizing alkaline effects of the free flow of saliva induced by the act of sucking. The condition called *furred tongue*, which was formerly considered as the result of a secretory process of the tongue itself, is regarded by modern science as essentially a growth of fungus, from vegetable spores deposited in the papillæ of the tongue from the food and from inspired air. The tongue being naturally cleaned by the friction of the teeth, if the latter are diseased and sore to the touch, the deposits upon the tongue remaining undisturbed are correspondingly increased.

Traumatic ulcers from the irritation caused by broken teeth frequently come under the notice of the dentist.

Ulcers formed under the tongue during whooping cough, and which were formerly supposed to be specific lesions of that disease, are probably due to friction from the teeth during the paroxysms. Amalgam fillings are sometimes supposed to cause apthous patches, but this probably is due to roughness of the fillings or margins, acting as a mechanical irritant. With the possibility of a specific parasite being always present, the dentist should, when apthous ulcers are present, use proper precaution not to carry the disease from one patient to another. Jagged teeth, rough fillings, and ill-fitting plates are enumerated among the exciting causes of cancer of the tongue, which is perhaps one reason why young persons and women are comparatively free from this form of cancer, the teeth of the former being sounder, and those of the latter better cared for than the teeth of men, friction from the pipe being another source of the disease in male adults.

The anterior half of the border of the tongue being most frequently affected with carcinomata points clearly to the teeth as the offending members. The slightest lesion of the tongue should be noted, and the source of irritation removed if possible. A very slight operation on the part of the dentist may sometimes prevent a simple ulcer from developing into a cancer of the tongue with its usually fatal termination.

*Dr. A. H. Thompson* read a second paper, entitled

PATHOLOGICAL HEREDITY AND GOUTY TEETH.

He said that it is a recognized fact in dental embryology that defects and deformities of the teeth are due to transmitted predisposition to certain diseases, though we are yet in the dark as to *how* this is effected. It is perhaps due to perversion of the nutritive fluid, the pabulum being poisoned by disease—or the formative organ itself may be affected, causing the death or distortion of the building cells. Although some authors attribute defective enamel to the action of acrid fluids at the time of eruption, the weight of evidence seems to be in favor of the congenital and follicular effects of disease, or, as Magitot says, the centre of the organ would remain unaffected. The markings and defects left by exanthemantic and intestinal affections are far less fatal to the teeth than the structural injuries due to hereditary influences. The effects of transmission are so invariable that certain characteristic marks are recognized pathognomonic signs of certain diseases, having a positive diagnostic value to the pathologist. Gout, syphilis, scrofula, scorbutis, tubercle, may be thus classed, though not yet fully proven. There are others of which we suspect much, though we as yet know positively but little.

Gout is not endemic in this country; errors in diet are here corrected by nature in other ways, so that the study of gout and gouty teeth is of less practical importance to American dentists than in the mother country. Yet they have a positive diagnostic value in determining the presence of hereditary gout. The disease depending probably upon an excess of uric acid may be either hereditary or acquired. The marks of gout upon the teeth have been carefully studied by D. Dyce Duckworth, Alf. Carpenter, W. Stewart, Horatio Doukin and others, the general verdict being that they are unusually large, strong, and regular and less subject to decay than usual. Children grind the teeth, even till adult life, chipping and breaking the teeth. They are usually yellow in tint and abnormally hard, but liable to wear down on articulating faces. Tartar accumulates in large quantities, men sometimes being found edentulous at the age of twenty-six.

*Dr. Carpenter* thinks gout not so marked in the teeth in early life as syphilis or tubercle.

The writer of the paper had had the opportunity of studying an imported case of genuine hereditary gout in the family of an English farmer, where gout was hereditary in both branches of the family for generations. The children therefore inherited directly from both parents. He exhibited casts of the teeth which were extremely characteristic. The teeth were short, with thick heavy shoulders, very dark in color and solid, dense, and hard in texture, with transverse or encircling grooves or step-like terraces. The gums were light and thin with sharp margins against the crown. The anterior teeth were all strongly marked, the first molars absent, the other molars normal. The history given of the extractions showed the difficulty belonging to this diathesis. Fothergill says there is little doubt of the distinctive diagnostic value of the configuration of gouty teeth, which are recognizable as soon as seen, being solid, massive, blunt, and thick at the edges, falling out without caries, the centre of the tooth darker in color from ostitis along the fangs of the incisors.

Discussion of subjects of Section VII now in order:

*Dr. Ingersoll* wished for a clearer definition of inherited disease.

*Dr. Thompson* said that he meant simply that it *was* inherited, or transmitted.

*Dr. Ingersoll* doubted that disease was ever transmitted. It was common to speak of consumption being inherited, but he doubted the propriety of the term. The diathesis or tendency to certain pathological conditions might be transmitted, as for instance a man who had a large wart developed at the age of forty years, and whose son had one exactly similar in exactly the same spot, as ascertained by measurement, developed at the same age, and which gave every promise of attaining the same dimensions, but the wart was not inherited : there was no wart there before he was forty years old, but the tendency was there. A whole family of children may be healthy through childhood and then all successively die at certain age of consumption, showing an inherited tendency to consumption, though there was no disease present in early life.

*Dr. Morgan* thought this distinction a mere play upon words. If a man has a certain temper and his son inherits it, it will usually be found to be something more than a mere tendency. The stamp of the character of the parent upon the children is, by stock men called potentiality, and depends upon the nervous rather than the physical organism. It requires a strong, positive, nervous temperament, or organism, to transmit unfailingly certain characteristics to posterity. (Here he cited the persistency of the "Morgan" type in horses, running through thirty-six years, and even now found in horses which have scarcely a drop of the original blood.)

With reference to the paper of Dr. Smith, he said that diseases of the oral cavity or of the tongue did not, as a rule, fall into the hands of dentists. In all of his long experience of forty years he had seen one, and only one, case where apthous patches did correspond with amalgam fillings, which being replaced with gold the ulcers disappeared. They were usually due to a depressed condition of the fluids of the body, though parasites were no doubt concerned also, as proved to his mind by their yielding to arsenical treatment.

*Dr. Brophy* thought that incipient cancer was more frequent than we suspected; he had seen a number in four years. They were aggravated, not so much by tobacco smoke as by the rough pipe-stem, though nicotine might also aggravate the trouble. He cited a case of epithelioma where four years ago a V shaped piece had been taken from the lower lip with no recurrence. Was satisfied that if it had been allowed to run on it would have developed in a carcinoma ending in death. He cited other cases, reaching the conclusion that if the operation was made early the chances were good for saving life, but if left alone this form of cancer developed very rapidly. He did not think any inherited tendency was concerned in these cases, or that there would have been any such development without the agency of the tooth or some other similar irritant. It was an assumption, a mere guess, with no means of positive knowledge. These affections are evidently on the increase, though writers say not. We see more cases, and hear of more.

*Dr. Ottofy* agreed with the paper of Dr. How, that the normal condition of the mouth and the oral fluids was a very important subject for the consideration of the dentist. He hoped the blanks of Dr. How would be taken hold of and careful investigations and records made.

*Dr. Harlan* said that with our present knowledge it seemed about settled that the explanation of much of the acid formation was to be found in the part played by germs in producing acid ferments. The recent experiments of Dr. Black seemed to establish the fact that acid ferments are the excrementitious products of germs. If they cause the acid saliva, what is the method of correction? It seems very simple: if the germs are destroyed we no longer have their products. Keep the mouth clean and free from decomposing food and other chemical processes, and free from germs and their products.

He would not attempt to discuss the paper of Dr. Thompson, as he had been too busy when it was read to give it proper attention.

The paper of Dr. Smith appeared to be very timely: too little attention was given to the condition of the tongue. It was subject to diseases, and was frequently a disgustingly dirty member. Patients should be taught how to keep the tongue clean, but they don't do it. Very few dentists attempt to treat lesions of the tongue; they say: it is not my province, the teeth bound my horizon. Attention not being directed to incipient lesions, the tongue or a portion of it, and even life itself, is lost.

The subject of the heredity of, or the transmission of tendency to, gout and gouty teeth in the United States is not of much practical importance to American dentists. They are too intensely occupied to acquire gout themselves; their means will not allow of it; they may get dyspeptic, but not gouty, and they are not likely to find many gouty teeth.

*Dr. Jas. Truman* said that his experience and observations were exactly the opposite to what had been stated. He did not, as a rule, find the mixed secretions of the oral cavity either alkaline or acid; they were habitually neutral. At the necks of the teeth, and places in a state of rest, the secretions were liable to be acid; during the night there was but little secretion of alkaline saliva, and then the secretions are acid. Tests are always made in the day, and the secretions usually found to be neutral. If they could be tested at night they would be found acid.

*Dr. Atkinson* said he was so happy he did not know where to begin. He wished to congratulate the Association on this presentment of scientific topics. As far as the science went, however, they were in the deepest kind of a muddle for lack of knowledge of the very alphabet of the process of nutrition and the elements of tissue; to make it clear would take more time and patience and preparation than can be given to it now.

Microbes are said to be the immediate cause of disease; they are the antecedents of unhealth. Living bodies die through retrogressive molecular changes, but we don't know *causes*; we only know antecedents and effects; I tremble when I hear men speak boldly of efficient causes! "Topeka" has seen the light, but he is all tangled up in his nomenclature. Protoplasm is the first plasm; there is only one protoplasm. Food is churned into pabulum, which is the food of all the tissues. If these things were better understood we should not hear it said, "the liver secretes bile!" bile is secreted *in* the liver. How do the molecules of the liquid water come from two gases? Was it before it was? It was *in esse*; an eternal entity, without beginning and without end, $H^2O$. What reduces the gases to a liquid?—the high priest awakening affinity in the molecules—and there again we get all tangled. Currents of electricity are awakened; water is the universal solvent producing saliva, gastric juice, chyme, chyle, blood, resolving all into pabulum, carried into needy territories and building up the body. Listening, in the mind, is the equivlent of the tissues waiting for pubulum. If we could but be delivered from false nomenclature!

Harlan had the cheek to tell me the first paper on pyorrhea had yet to be written; the child was born well, but he has not been educated up; give him ten years by the side of our blessed old Secretary to hold him to his duty, and he would grow to be a giant!

When he takes one breath, he wants to blow it out as fast as possible, and get rid of the ashes and lye. If well bleached of lyes (lies?) he would be a light on a hill that could not be hid, but you have all been falsely educated and will not receive the inspiration that comes home to your consciences.

*Dr. Thompson* said, in reply to Dr. Ingersoll, that he did not see the value of the distinction drawn between inherited disease or transmitted idiosyncrasy or diathesis.

It made little difference whether we said the disease was transmitted, or the diathesis was transmitted as an impression, so long as children were born syphilitic; he only wished to bring to notice a certain form of idiosyncrasy.

On motion, subject passed.

*Dr. Crouse* offered a report from the executive committee, giving a history of the difficulties encountered in the choice of place of meeting, and recommending several changes in the Constitution which should define the duties of the executive committee so clearly that there could be no future mistakes. After some heated discussion, certain portions of the report were ordered stricken out and the remainder accepted.

The resolutions to amend the Constitution being objected to, lie over till next year.

The Committee on Credentials reported a delegate present from the New Jersey State Dental Society.

On motion, adjourned to 9 A.M.

FRIDAY, AUG. 6—FOURTH DAY—FIRST SESSION.

Called to order at 9:20 A.M., the President in the Chair.

The minutes of the last session were read and approved.

The President announced the following as the committee of three to confer with manufacturers of mineral teeth: Drs. L. P. Haskell, W. B. Ames, and W. H. Truman.

*Dr. C. N. Pierce* introduced a resolution discountenancing by the Association the customary banquets and entertainments during or at the close of the meeting, the Society being too large to be entertained at the expense of any local society.

Unanimously adopted.

*Dr. S. W. Dennis*, of San Francisco, Cal., introduced a resolution that it be considered the sense of the Association that the time and place of meeting of the different professional and scientific associations be made as nearly uniform as possible, in order that members from different portions of the country may be enabled to attend them all in close succession, and that copies of this resolution be sent to all such societies.

After some discussion by Drs. Kingsley, Abbott, Atkinson, Guilford, Dennis, and others, the motion was lost.

*Dr. Marshall* moved that the Association recommend to the various dental colleges that in obediance to the demands of higher education their sessions be uniformily extended to not less than nine months in each year.

Referred to the Committee on Education.

On motion of Dr. Atkinson, Dr. E. A. Bogne was accredited as the delegate from the American Dental Association to the British Dental Association, and the German Medical and Scientific Association, which will shortly hold their annual sessions.

*Dr. Brophy* stated that Sections II. and III., Dental Education, and Dental Literature and Nomenclature desired to be consolidated.

Unanimous consent was given, and Section II. formed from the old Sections II. and III.

*Dr. Marshall* asked that the new section authorized by a previous resolution, be known as Section III., and be known as Pathology and Surgery.

*Dr. Harlan*, chairman of Section VI., stated that this change would greatly facilitate the work of Section VI., which is too large.

Unanimously adopted, and Section VI. of Constitution so amended.

The selection of place of next meeting being the next order of business, the following places were put in nomination:

Chicago, Ill.
Old Point Comfort, Va.
Asheville, N. C.
Asbury Park, N. J.
Salt Lake City, Utah.
Chautauqua, N. Y.
Louisville, Ky.
Crescent Springs, Pa.

Drs. Crouse and Kingsley spoke for Salt Lake City; Dr. G. W. McIlhaney for Asheville; Dr. Guilford for Old Point Comfort; Dr. Lundenburg for Crescent Springs; Prof. Truman and Dr. Levy spoke for Asbury Park.

*Dr. Abbott* objected to this place as being in New Jersey, and not big enough to hold the Association.

*Dr. Kingsley* said there was plenty of room, but it was a temperance town.

*Dr. Parmley Brown* hoped they would go South.

*Dr. Dennis* spoke strongly in favor of San Francisco, and mentioned the great reduction in railroad rates as compared with 1870, when only three more votes were wanted to carry the body to the Pacific Coast, and extended a most cordial invitation on the part of the Odontological Society of San Francisco. The President also read a telegram received to that effect.

Drs. Abbott and McManus were appointed tellers.

On the second ballot, ASHVILLE, N. C., received a very large majority of the votes cast, and the vote was made unanimous.

*Dr. Thompson* called attention to the fact that the resolution changing the time of meeting to the last Tuesday in August was laid over until the next meeting. The Executive Committee could however change the time in case of emergency.

On the informal ballot for nomination for President, Dr. W. W. Allport, Chicago, received forty-nine votes, Dr. R. B. Winder, twenty-seven, and Dr. Ingersoll, seven, with a number of scattering votes.

Dr. Allport having received a majority the informal vote was made formal, and Dr. Allport announced as President.

*Dr. G. W. McElhaney* (Columbus, Ga.) was elected first Vice-President.

*Dr. S. W. Dennis* (San Francisco), Second Vice-President.

*Dr. Geo. H. Cushing*, Recording Secretary,

*Dr. A. W. Harlan*, Corresponding Secretary,

*Dr. Geo. W. Keeley*, Treasurer; the latter three were re-elected.

Drs. W. C. Wardlaw (Augusta, Ga.,) T. G. Perry (New York), and S. H. Guilford (Philadelphia), were elected members of the Executive Committee in place of Drs. Morrison, Fredrichs, and Perry, whose term expired.

Dr. Crouse and Dr. C. N. Pierce resigned from the Executive Committee.

Drs. E. T. Darby (Philadelphia) and A. W. Harlan (Chicago), were elected to fill their unexpired terms.

A vote of thanks was tendered the railroads which had given reduced return fare, the hotels which had given reduced rates, the press for courtesies extended.

On motion of Dr. H. A. Smith, a resolotion was offered and carried, inviting the Southern Dental Association to meet with the American in joint session at Asheville, N. C.

*Dr. A. O. Hunt* moved the renewal of the appropriations to Sections V., VI., and VII. for carrying on scientific work during the year.

*Dr. Marshall* stated that the money appropriated to Section VI. had not been expended, but the plans laid for the ensuing year, would require it.

The President decided that money appropriated for work in 1886 remaining unexpended, should be covered back into the Treasury, new appropriations being made for 1887.

*Dr. Marshall* said that was very unfortunate—not having used the $200.00 allowed them, their plans of work for the coming year based on the expectation of having $400.00 to use.

*Dr. Sitherwood* said that Section III. (now combined with II.) desired an appropriation of not more than $200.00 for the purchase of the latest literature for the use of the committee.

Not acted on.

The roll of sections was called once more. Sections II. and III. not having offered any report. These sections had no formal report to make and no papers had been offered. The other sections had no further reports to offer.

The final minutes were read and declared correct.

*Dr. W. C. Barrett*, the retiring President, thanked the Association for their kind consideration. He said he had doubtless made mistakes, but God knew they were mistakes of the head, not of the heart. It would always be the pride of his life to remember that he had been the President of the American Dental Association.

He then appointed Drs. Frank Abbott and J. Taft to conduct the President elect to the chair, and retired with dignity from the chair he had so ably filled.

Dr. Barrett was presented with the handsome ivory gavel he had wielded so judiciously, which was ordered suitably inscribed.

He then introduced his successor, Dr. W. W. Allport, to the kind consideration of the Association, hoping he would meet with as little of the unpleasantness of official position as he had done.

President Allport accepted the position with brief but appropriate words. He said he was neither unmindful of the honor, nor ignorant of the responsibilities he was accepting. Looking back at the record of the Association for a quarter of a century, he asked that any and all unpleasantness be now forgotten and left with the past, and hoped that all their proceedings would be such as to add to the good name and increase the influence of the profession, and of that individual reputation which constitutes much of the little we have to leave to our children. He could only promise that he would do all in his power to discharge his duties acceptably.

As his first official act he appointed as the publishing committee, Dr. E. T. Darby and Dr. A. W. Harlan (the secretary is ex-officio a member of that committee). As the local committee of arrangements, Dr. Douglass, Ashville, Dr. T. T. Moore, Columbia, S. C., and Dr. Bland, N. C.

The other newly elected officers briefly expressed their appreciation of the honor conferred—the Treasurer waving above his head a *greenback* as the expression of his sentiments.

After reading the last items of the final minutes, adjourned, to meet at Ashville, N. C., on the first Tuesday in August, 1887.

